# Assessment of Diagnostic Yield of Nonculture Infection Testing on Cerebrospinal Fluid in Immune-Competent Children

**DOI:** 10.1001/jamanetworkopen.2019.7307

**Published:** 2019-07-19

**Authors:** Jennifer L. McGuire, Nichole V. Tuite, Sanjeev K. Swami, Robert A. Avery

**Affiliations:** 1 Division of Neurology, The Children’s Hospital of Philadelphia, Philadelphia, Pennsylvania; 2 Department of Pediatrics, The Perelman School of Medicine at the University of Pennsylvania, Philadelphia; 3 Department of Neurology, The Perelman School of Medicine at the University of Pennsylvania, Philadelphia; 4 Division of Infectious Disease, The Children’s Hospital of Philadelphia, Philadelphia, Pennsylvania; 5 Division of Ophthalmology, The Children’s Hospital of Philadelphia, Philadelphia, Pennsylvania; 6 Department of Ophthalmology, The Perelman School of Medicine at the University of Pennsylvania, Philadelphia

## Abstract

**Importance:**

Nonculture infection tests of cerebrospinal fluid (CSF) samples using polymerase chain reaction and antigen or antibody assays are frequently ordered on lumbar puncture specimens concurrently with routine CSF cell counts, but the value of CSF infection testing in otherwise healthy children is unknown.

**Objective:**

To determine the value of nonculture CSF infection testing in immune-competent children with normal CSF cell counts.

**Design, Setting, and Participants:**

This cross-sectional study reviewed screening and diagnostic tests in the electronic medical record system of a large academic tertiary care children’s hospital. Records of children aged 0.5 to 18.9 years (n = 4083) who underwent lumbar puncture (n = 4811 procedures) in an inpatient or outpatient facility of Children's Hospital of Philadelphia between July 1, 2007, and December 31, 2016, were reviewed. Those with indwelling CSF shunts or catheters; those with active or past oncologic, immunologic, or rheumatologic conditions; or those taking immune-suppressing medications were excluded from analysis. This study was conducted from July 20, 2017, to March 13, 2019.

**Main Outcomes and Measures:**

Outcome variables included frequency of nonculture CSF infection testing and frequency of positive results in the entire cohort, and among those with normal cell counts. Normal cell counts were defined as CSF white blood cell counts lower than 5 cells/μL and CSF red blood cell counts lower than 500 cells/μL.

**Results:**

In total, 4811 lumbar puncture procedures were performed on 4083 unique children, with a median (range) age of 7.4 (0.5-18.9) years, 2537 boys (52.7%), and 3331 (69.2%) with normal CSF cell counts. At least 1 nonculture CSF infection test was performed on 1270 lumbar puncture specimens with normal cell counts (38.1%; 95% CI, 36%-40%), and more tests were performed in the summer months. Only 18 (1.4%; 95% CI, 0.9%-2.2%) of 1270 lumbar puncture specimens with normal cell counts had at least 1 nonculture infection test with a positive result; 2 of these 18 children required clinical intervention for their positive results, but each also had other clear clinical signs of infection.

**Conclusions and Relevance:**

Nonculture CSF infection testing appeared to be common in immune-competent children with normal CSF cell counts, but positive results were uncommon and were not independently associated with clinical care; delaying the decision to send nonculture infection tests until CSF cell counts are available could reduce unnecessary diagnostic testing and medical costs, which may improve value-based care.

## Key Points

**Question:**

What is the value of nonculture cerebrospinal fluid infection testing in immune-competent children with normal cerebrospinal fluid cell counts?

**Finding:**

In this cross-sectional study of 4811 lumbar puncture specimens from immune-competent children aged 0.5 to 18.9 years, only 2 specimens with positive results from nonculture cerebrospinal fluid infection testing required clinical intervention, among all specimens with normal cell counts. Both children who required clinical intervention had other clinical signs of infection.

**Meaning:**

Nonculture cerebrospinal fluid infection testing appeared to be common in immune-competent children with normal cerebrospinal fluid cell counts, but positive results seemed rare and unlikely to be independently associated with care.

## Teaser

This cross-sectional study examines the value of nonculture cerebrospinal fluid (CSF) testing in immune-competent children with normal CSF cell count.

## Introduction

Acute central nervous system (CNS) infections, such as meningitis and encephalitis, require prompt recognition of treatable etiologies (eg, bacterial pathogens, herpes simplex virus [HSV]) to reduce potentially significant morbidity and mortality. Infections of the CNS are typically identified with lumbar puncture and evaluation of cerebrospinal fluid (CSF) cell counts, protein, glucose, Gram stain, bacterial culture, and nonculture infection tests. A CSF white blood cell (WBC) count lower than 5 cells/μL in immune-competent children older than 2 months is considered normal, whereas a count of 5 cells/μL or higher suggests infection and/or inflammation^1^ (to convert to ×10^9^ per liter, multiply by 0.001). The presence of red blood cells (RBCs) in CSF samples represents either intrathecal blood (eg, subarachnoid or intraparenchymal hemorrhage) or introduction of peripheral blood into CSF during the performance of the lumbar puncture. Introduction of peripheral blood into otherwise sterile CSF may result in the potential contamination of a bloodstream pathogen, and it also makes the CSF WBC difficult to clinically interpret because an elevated count may reflect a true pleocytosis and/or peripheral blood WBCs.

Polymerase chain reaction (PCR) of CSF and antigen or antibody assays are used to identify specific pathogens in suspected CNS infection; however, their overall diagnostic yield is variable. Nonculture CSF infection test sensitivity and specificity are high for some organisms (eg, HSV PCR,^2^ enterovirus PCR^3^) but are low for others (eg, *Borrelia burgdorferi* PCR and antibody assays^4^). The positive and negative predictive values, as well as the clinical advantages of these tests in populations with a low pretest probability for CNS infection (such as immune-competent children with normal CSF cell counts), are unknown.

Laboratory testing with little clinical advantage but a disproportionately high cost is one example of low-value care. When a lumbar puncture is performed, frontline clinicians often order nonculture CSF infection tests concurrently with CSF cell counts, protein level, glucose level, and Gram stain to optimize administrative workflow and minimize the potential need later to collect more CSF. Even though lumbar puncture has a low complication rate,^5,6^ it often requires sedation for children, making a repeated procedure undesirable. To determine if ordering practices and laboratory workflow for nonculture CSF infection tests may be a target to reduce low-value care in pediatrics, we sought to identify the frequency of positive nonculture CSF infection tests and their association with the clinical care of immune-competent children with normal CSF cell counts.

## Methods

We reviewed the electronic medical record (EMR) of Children’s Hospital of Philadelphia, a large academic tertiary care children’s hospital. The Children’s Hospital of Philadelphia Institutional Review Board approved this study and waived the informed consent requirement and Health Insurance Portability and Accountability Act authorization because the research and use of personal health information poses no greater than minimal risk and the study could not be performed without the waiver, which did not adversely affect the rights or welfare of the patients. This study followed the Strengthening the Reporting of Observational Studies in Epidemiology (STROBE) reporting guideline for cross-sectional studies. The study was conducted from July 20, 2017, to March 13, 2019.

We queried the EMR for any CSF cell count performed on a child aged 0.5 to 18.9 years at an inpatient or outpatient Children’s Hospital of Philadelphia facility between July 1, 2007, and December 31, 2016. Some children underwent more than 1 lumbar puncture during this study period and contributed multiple independent data points over time to the analysis. We defined exclusion criteria according to *International Classification of Diseases, Tenth Revision,* codes in the EMR at or before the time of the lumbar puncture. We excluded children (1) with indwelling CSF shunts or catheters; (2) with malignant oncologic, immunologic, or rheumatologic diagnosis that may have interfered with the ability to mount an appropriate pleocytosis in response to the infection at the time of the lumbar puncture; (3) who underwent lumbar puncture in an oncology department, when the location of the procedure was available; and (4) with EMR documentation of a medication, taken at the time of the lumbar puncture, known to suppress the immune system.

Patient data, including medical record number, demographic characteristics, epidemiologic information, and CSF test results were extracted from the EMR on children who met the inclusion and exclusion criteria. These data were manually reviewed in the EMR to verify suspected errors and missing data, confirm the accuracy of extracted data (all nonculture CSF infection tests were manually reviewed for lumbar puncture specimens with normal cell counts), and develop an abstract of clinical data from specimens with normal cell counts and a positive nonculture infection test result.

### Study Definitions

Normal cell counts were defined as CSF WBC lower than 5 cells/μL and CSF RBC lower than 500 cells/μL; elevated cell counts were defined as CSF WBC 5 cells/μL or higher and/or CSF RBC 500 cells/μL or higher. When more than 1 set of cell counts were obtained for a given lumbar puncture specimen, we used the lowest value of each WBC and RBC count. We recorded CSF protein and glucose values less than a lower limit of detection (eg, CSF glucose <10 mg/dL [to convert to millimoles per liter, multiply by 0.0555]) as the lower limit of detection.

The *International Classification of Diseases, Tenth Revision*, codes used to define exclusion criteria are summarized in the eMethods in the Supplement. Corticosteroids were not included as an exclusionary immune-suppressing medication given the varying immune outcomes by medication and dosing by body weight.

Nonculture CSF infection tests we examined were PCR, antibody testing (IgG and/or IgM), and antigen testing. We included these tests in the analysis only if they were ordered on the same day that CSF cell counts were obtained; tests ordered on an existing specimen at a later date were excluded. Specific monoplex, real-time, qualitative CSF PCR tests we examined were those for the following: adenovirus (laboratory developed test [LDT], using published primer and probe sequences),^7^ *Borrelia burgdorferi* (sent to Associated Regional and University Pathologists [ARUP] Laboratories), cytomegalovirus (LDT, using published primer and probe sequences),^8^ Epstein-Barr virus (LDT, using published primer and probe sequences),^9^ enterovirus (LDT, using published primer and probe sequences),^10^ human herpesvirus 6 (HHV-6; LDT, using published primer and probe sequences),^11^ herpes simplex virus (HSV; LDT, using published primer and probe sequences),^12^ human parechovirus (LDT, using published primer and probe sequences),^13^ influenza A and B virus (LDT, based on communication with Centers for Disease Control and Prevention), human metapneumovirus (LDT, using published primer and probe sequences),^14^ mumps virus (sent to the Pennsylvania state laboratory), *Mycoplasma pneumoniae* (LDT, using published primer and probe sequences),^15^ parainfluenza virus 1-3 (LDT, using published primer and probe sequences),^16,17^ parvovirus B19 (LDT, using published primer and probe sequences),^18^ varicella zoster virus (VZV; LDT, using published primer and probe sequences),^19^ and West Nile virus (sent to ARUP Laboratories).

The Children’s Hospital of Philadelphia does not use commercial multiplex PCR tests for CSF samples because of performance concerns.^20^ Antigen or antibody CSF testing included arbovirus IgG or IgM panels (testing for California encephalitis, Eastern equine encephalitis, St Louis encephalitis, Western equine encephalitis, and West Nile virus; sent to ARUP Laboratories), cryptococcal antigen (ran in house [lateral flow assay; Immuno Mycologics]), Epstein-Barr virus IgG panels (ran in house [Zeus Scientific]), lymphocytic choriomeningitis virus IgG or IgM (sent to ARUP Laboratories), Lyme total antibody level (to define if someone was tested for Lyme; sent to ARUP Laboratories), Lyme Western blot IgG or IgM (to define a positive Lyme test result; sent to ARUP Laboratories), measles IgG or IgM (sent to Pennsylvania Department of Health), rubella IgG or IgM (IgG ran in-house [Captia assay; Trinity Biotech]; IgM sent to ARUP Laboratories), toxoplasmosis IgG or IgM (IgG ran in-house [Captia assay; Trinity Biotech]; IgM sent to ARUP Laboratories), VZV IgG or IgM (IgG ran in-house [Captia assay; Trinity Biotech]; IgM sent to ARUP Laboratories), and venereal diseases research laboratory assay (sent to ARUP Laboratories).

Nonculture CSF infection test results were recorded as positive, negative, or not performed. Medical records with either IgG- or IgM-positive antibody CSF test results were manually reviewed to determine the clinical interpretation as positive or negative for the child’s clinical presentation. We manually reviewed all indeterminate or equivocal antibody testing results in the EMR, and if repeated confirmatory testing was not performed, we conservatively considered the results positive. When any component of a multitest panel (eg, West Nile virus IgM in an arbovirus panel) had a positive result, we considered the overall panel results positive.

### Statistical Analysis

Data were analyzed using Stata, version 14.2 (StataCorp LLC). Nonparametric methods were used because not all continuous variables (such as age) were normally distributed and to minimize the effect of outliers with statistical associations. Continuous variables were described using medians and interquartile ranges (IQRs), and intergroup differences were compared using Wilcoxon rank sum tests. Categorical variables were described using counts and frequencies, and intergroup differences compared using the χ^2^ test. Statistical significance was determined a priori as a 2-tailed *P* < .05.

After the overall cohort was analyzed, the subset of infants aged 6 to 12 months was also examined separately, given our a priori concern that their relatively immature immune system could potentially be a factor in the test results being different from those of older children.

## Results

Cohort demographics and key laboratory characteristics of the overall cohort and subgroups by cell counts (normal and elevated) are summarized in Table 1. Overall, 4811 lumbar puncture procedures were performed on 4083 unique children, with a median (range) age of 7.4 (0.5-18.9) years and 2537 (52.7%) of whom were boys. The distribution of age was skewed to the left, with 1161 (24.1%) of 4811 procedures performed in children younger than 2 years. Children with elevated CSF cell counts were more often male (825 [55.7%] vs 1712 [51.4%]; *P* = .005) and African American (519 [35.1%] vs 975 [29.3%]; *P* = .001) compared with children with normal CSF cell counts (Table 1); median age did not differ between groups.

**Table 1.** Characteristics of All Children Who Underwent Lumbar Puncture Procedure

Variable	All (n = 4811)	CSF Cell Counts		
		Elevated (n = 1480)	Normal (n = 3331)	*P* Value^a^
Age, median (IQR), y	7.4 (2.1-13.8)	7.2 (2.2-12.9)	7.5 (2.0-14.1)	.06
Male, No. (%)	2537 (52.7)	825 (55.7)	1712 (51.4)	.005
African American, No. (%)	1494 (31.1)	519 (35.1)	975 (29.3)	.001
CSF parameters, median (IQR)				
Glucose, mg/dL^b^	55 (49-64)	53 (47-63)	55 (50-64)	<.001
Protein, g/dL^c^	24 (16-38)	43 (26-88)	20 (14-29)	<.001
CSF testing practices				
Any CSF pathogen-directed test sent, No. (%)	2038 (42.4)	768 (51.9)	1270 (38.1)	<.001
>1 Test sent, No./total No. (%)	1368/2038 (67.1)	541/768 (70.4)	827/1270 (65.1)	.01
Tests sent, median (range), No.	2 (1-11)	2 (1-10)	2 (1-11)	.02
CSF testing yield				
All ages, No./total No. (%)				
Any nonculture infection test from LP with positive result	227/2038 (11.1)	209/768 (27.2)	18/1270 (1.4)	<.001
Infants, aged 6-11.9 mo				
Any nonculture infection test from LP with positive result	13/169 (7.7)	10/54 (18.5)	3/115 (2.6)	<.001

Abbreviations: CSF, cerebrospinal fluid; IQR, interquartile range; LP, lumbar puncture.

SI conversion factors: To convert glucose to mmol/L, multiply by 0.0555; protein to g/L, multiply by 10.0.

^a^ *P* values to compare characteristics between those with elevated and those with normal cell counts were calculated using χ^2^ tests for categorical variables and Wilcoxon rank sum tests (medians) for continuous variables.

^b^ Data available for 4647 children (1374 with elevated cell counts, and 3273 with normal cell counts.

^c^ Data available for 4644 children (1376 with elevated cell counts, and 3268 with normal cell counts).

**Figure 1.**
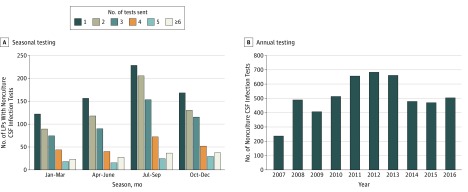
Nonculture Cerebrospinal Fluid (CSF) Infection Testing Frequency Over Time Testing frequency did vary by season but has been consistent annually for at least the past 9 to 10 years. LP indicates lumbar puncture.

Among the total 4811 lumbar puncture specimens, 2038 (42.4%; 95% CI, 41%-44%) had at least 1 nonculture CSF infection test sent: 270 specimens had both PCR and antibody or antigen testing, 1613 had only PCR testing, and 155 had just antibody or antigen testing. Among those specimens that had at least 1 nonculture CSF infection test, 1368 (67.1%; 95% CI, 65%-69%) had more than 1 test (median [range], 2 [1-11]). Testing was more frequent in the summer months compared with the remainder of the year (Figure 1A) but was consistent annually overall (Figure 1B). Only 227 (11.1%; 95% CI, 10%-13%) of the 2038 lumbar puncture specimens that had nonculture CSF infection testing, or 229 (4.5%; 95% CI, 3.8%-5.1%) of the 5054 total nonculture infection tests performed on these specimens, had a positive result. Enterovirus PCR (n = 186) accounted for 81% (95% CI, 76%-86%) of all nonculture CSF infection tests with a positive result, followed by Lyme Western blot (n = 12), HSV PCR (n = 10), HHV-6 PCR (n = 7), VZV PCR (n = 7), VZV IgM (n = 1), and others (Table 2).

**Table 2.** Frequency of Nonculture Cerebrospinal Fluid Infection Tests and Positive Yield

Test	All Tests (n = 4481)		Normal Cell Counts (n = 3331)	
	Ordered, No.	Positive Result, No. (% Ordered)	Ordered, No.	Positive Result, No. (% Ordered)
Enterovirus PCR	1481	186 (12.6)	869	12 (1.4)
HSV PCR	1214	10 (0.8)	774	1 (0.1)
Parechovirus PCR	779	0 (0)	461	0 (0)
Lyme total antibody/Western blot^a^	297	12 (4.0)	177	0 (0)
HHV-6 PCR	277	7 (2.5)	177	3 (1.7)
VZV PCR	234	7 (2.9)	124	0 (0)
Lyme PCR	150	0 (0)	133	0 (0)
EBV PCR	132	1 (0.8)	69	0 (0)
WNV PCR	103	0 (0)	44	0 (0)
CMV PCR	87	0 (0)	45	0 (0)
Cryptococcal antigen	86	0 (0)	38	0 (0)
Adenovirus PCR	69	0 (0)	30	0 (0)
Mycoplasma PCR	41	0 (0)	21	0 (0)
VZV IgM	28	1 (3.6)	18	0 (0)
VDRL	17	0 (0)	13	0 (0)
Parvovirus B19 PCR	15	0 (0)	8	0 (0)
Arbovirus IgG/IgM panel	14	2 (14.3)	8	0 (0)
Influenza A PCR	4	0 (0)	1	0 (0)
Influenza B PCR	4	0 (0)	2	0 (0)
Measles IgG/IgM	4	2 (50.0)	4	2 (50.0)
Mumps PCR	2	0 (0)	0	0 (0)
Parainfluenza type 3 PCR	2	0 (0)	0	0 (0)
EBV IgG/IgM panel	2	0 (0)	0	0 (0)
LCMV IgG/IgM	7	0 (0)	2	0 (0)
VZV IgG	3	0 (0)	2	0 (0)
HMPV PCR	1	0 (0)	0	0 (0)
Toxoplasmosis IgG/IgM	1	1 (100)	1	1 (100)
Total tests	5054	229 (4.5)	3021	19 (0.6)

Abbreviations: CMV, cytomegalovirus; EBV, Epstein-Barr virus; HHV-6, human herpesvirus 6; HMPV, human metapneumovirus; HSV, herpes simplex virus; IgG, immunoglobulin G; IgM, immunoglobulin M; LCMV, lymphocytic choriomeningitis virus; PCR, polymerase chain reaction; VDRL, venereal disease research laboratory; VZV, varicella zoster virus; WNV, West Nile virus.

^a^ Tests ordered defined by all Lyme total antibody assay. Percentage of positive results determined by reflex Western blot test.

**Figure 2.**
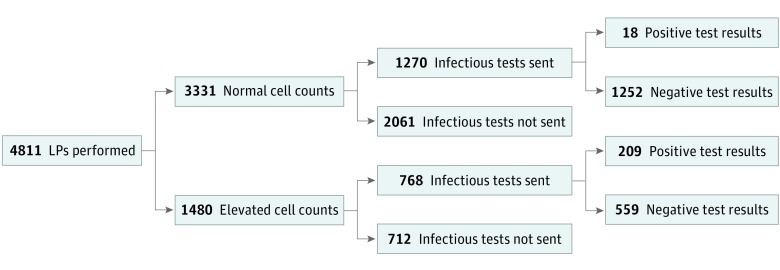
Flow Diagram Lumbar puncture (LP) specimens with normal cell counts were common in immune-competent children of all ages but were rarely associated with a positive result from a nonculture cerebrospinal fluid infection test.

Of the 4811 lumbar puncture procedures performed, 3331 specimens (69.2%; 95% CI, 68%-71%) performed on 3033 unique children had normal CSF cell counts, of which 1270 (38.1%; 95% CI, 36%-40%) had at least 1 sent for nonculture CSF infection test. Among those 1270 specimens with normal cell counts sent for a nonculture CSF infection test, 827 (65.1%; 95% CI, 62%-68%) were sent for more than 1 test (median [range], 2 [1-11] tests). Only 18 (1.4%; 95% CI, 0.9%-2.2%) of 1270 specimens with normal cell counts tested, or 19 (0.6%; 95% CI, 0.4%-1%) of the 3021 total nonculture CSF infection tests performed on these specimens, had a positive result (Figure 2). Positive CSF test results in this subgroup included enterovirus PCR (n = 12), HHV-6 PCR (n = 3), HSV PCR (n = 1), measles IgG (n = 2), and toxoplasmosis IgG (n = 1). The 2 children who each had positive antibody testing also had recently received intravenous immunoglobulin, which may have confounded positive test results. Overall, positive results had active treatment implications for only 2 children. A 2-year-old with Hunter syndrome (mucopolysaccharidosis type II) had a positive CSF HSV PCR result and a magnetic resonance imaging scan highly suggestive of HSV encephalitis. An 11-year-old had positive results for CSF measles IgG and toxoplasmosis IgG; the latter was believed to be a false-positive associated with intravenous immunoglobulin. The child presented with progressive neurologic deterioration and a profoundly abnormal electroencephalogram and magnetic resonance imaging scan and ultimately received a diagnosis of subacute sclerosing panencephalitis (Table 3).

**Table 3.** Clinical and CSF Characteristics of Children With Normal Cell Counts and Nonculture CSF Infection Testing With a Positive Result

Patient No.	Age^a^	WBC Count, No.	RBC Count, No.	Protein, mg/dL	Glucose, mg/dL	Positive-Result CSF Infection Test	Clinical Notes
1	Adult	1	8	16	57	CSF enterovirus PCR	Previously healthy. New frontal headache, emesis, and ataxia. Admitted. HCT normal. Empirical antibiotics for 24 h, not affected by enterovirus PCR.
2	Child	1	0	49	36	CSF enterovirus PCR	Spastic quadriparesis and ventilator-dependent tracheostomy after motor vehicle crash at age 2 y. New altered mental status in setting of gastrointestinal illness. Admitted. HCT/MRI stable. Empirical acyclovir sodium and antibiotics for 24 h, not affected by enterovirus PCR.
3	Child	2	0	18	47	CSF enterovirus PCR	Previously healthy. New-onset headache, nausea, and photophobia with pain on neck flexion. HCT normal. Symptoms treated. Discharged home prior to diagnosis.
4	Child	1	11	11	50	CSF enterovirus PCR	Previously healthy. New-onset headache and fever. Discharged home prior to diagnosis.
5	Child	1	71	20	53	CSF enterovirus PCR	Previously healthy. New-onset headache and fever. Discharged home prior to diagnosis.
6	Child	3	1	15	61	CSF enterovirus PCR	Previously healthy. New-onset headache and emesis, and possible altered mental status. HCT normal. Discharged home prior to diagnosis.
7	Child	2	14	19	110	CSF enterovirus PCR	History of neonatal seizures. Prolonged 45-min focal seizure and respiratory failure. HCT normal, and MRI stable. Discharged home prior to diagnosis.
8	Child	2	0	17	86	CSF enterovirus PCR	Previously healthy. New-onset fever and seizure. Admitted. Empirical acyclovir and antibiotics for 48 h, not affected by enterovirus PCR.
9	Child	2	2	12	68	CSF enterovirus PCR	Previously healthy. New-onset altered mental status. Admitted. HCT normal. Acyclovir stopped when enterovirus PCR test result was returned positive.
10	Child	2	2	16	87	CSF enterovirus PCR	Previously healthy. New-onset status epilepticus. Admitted. Empirical acyclovir and antibiotics, not affected by enterovirus PCR.
11	Infant	1	150	29	57	CSF enterovirus PCR	Previously healthy. New intermittent unresponsiveness, and tensing extremities. Discharged home prior to diagnosis.
12	Infant	2	34	24	54	CSF enterovirus PCR	Previously healthy. New seizure in context of rhinorrhea, cough, and fever. Diagnosed febrile seizure. Discharged home prior to diagnosis.
13	Child	3	0	40	43	CSF HHV-6 PCR	34 4/7-wk estimated gestational age, premature infant with recent meningoencephalitis (CSF WBC 270 cells/μL), treated with acyclovir for 21 d, and presumed HSV but test result negative. New recurrent papular rash, headache, and parental concern for relapse. Immunology consulted for possible immune deficiency. Discharged home prior to diagnosis.^b^
14	Child	0	0	27	85	CSF HHV-6 PCR	Previously healthy twin. Six wk of emesis and diarrhea, new lethargy. Admitted. HCT normal. Empirical antibiotics for 48 h. Positive result of serum HHV-6 PCR, clinical syndrome consistent with roseola. CSF HHV-6 PCR ordered for confirmation of HHV-6 encephalitis, did not affect antibiotic course.
15	Infant	1	0	10	69	CSF HHV-6 PCR	35-wk estimated gestational age premature infant. New seizure and fever. Diagnosed complex febrile seizure. Empirical antibiotics for 48 h, not affected by HHV-6 PCR.
16	Child	1	240	36	70	CSF measles IgG and toxoplasmosis IgG	Previously healthy. New abnormal MRI with diffuse T2 hyperintensity in subcortical and deep white matter, and abnormal EEG. Previous treatment with IVIg. Diagnosed with SSPE per diagnostic criteria^21^^c^
17	Child	3	30	20	46	CSF measles IgG	Previous diagnosis of X-linked agammaglobulinemia, on monthly IVIg. New left-sided clumsiness, which triggered outpatient MRI that was abnormal. LP performed as outpatient in follow-up of MRI findings. No clinical action taken on this positive laboratory test result, thought related to previous IVIg.^b^
18	Child	1	0	31	72	CSF HSV PCR	Previous diagnosis of Hunter syndrome (mucopolysaccharidosis type II). New focal seizures. MRI concerning for HSV encephalitis.^c^

Abbreviations: CSF, cerebrospinal fluid; EEG, electroencephalogram; HCT, head computed tomography; HHV-6, human herpesvirus 6; HSV, herpes simplex virus; IgG, immunoglobulin G; IVIg, intravenous immunoglobulin; LP, lumbar puncture; MRI, magnetic resonance imaging; PCR, polymerase chain reaction; RBC, red blood cell; SSPE, subacute sclerosing panencephalitis; WBC, white blood cell.

SI conversion factors: To convert glucose to mmol/L, multiply by 0.0555; protein to g/dL, divide by 1000.

^a^ Infant indicates ages 1 month to 1 year; child, 1 to 12 years; and adult, 18 years and older.

^b^ Previous diagnosis of immunodeficiency not captured by electronic medical record filters (X-linked agammaglobulinemia) or by clinical concern outlined in notes.

^c^ Test results resulted in specific therapy change in the plan of care.

To explore the role of CSF RBC values in these data, we performed a sensitivity analysis to include children with elevated CSF RBC in the original normal cell count subgroup. This inclusion added 191 lumbar puncture specimens to the sensitivity analysis, for a total of 3522 specimens. Among these 3522 specimens with normal CSF WBC alone, 1348 (38.3%; 95% CI, 37%-40%) were sent for at least 1 nonculture CSF infection test, of which 22 (1.6%; 95% CI, 1.1%-2.5%) had a positive result. However, none of the additional 4 procedures with positive results (0 HSV, 1 enterovirus PCR, and 3 HHV-6 PCR) had active treatment implications.

Of the 4811 lumbar puncture procedures that met inclusion and exclusion criteria, 537 (11.2%) were performed on 460 unique infants between 6 and 12 months of age. Among the 537 procedures performed on these infants, 169 (31.5%; 95% CI, 28%-36%) had specimens sent for at least 1 nonculture CSF infection test, for a total of 352 nonculture infection tests. Only 13 (7.7%; 95% CI, 4.2%-12.8%) of the 169 lumbar puncture procedures performed on infants who had nonculture CSF infection testing, or 13 (3.7%; 95% CI, 2.0%-6.2%) of the 352 total nonculture infection tests performed on these procedures, had a positive result. These proportions are each slightly lower than seen in the overall cohort. Three hundred fifty-one (65.4%; 95% CI, 61%-69%) of 537 lumbar puncture specimens in infants had normal cell counts, a similar proportion to the overall cohort. One hundred fifteen (32.8%; 95% CI, 28%-38%) of 351 lumbar puncture specimens in infants with normal cell counts had at least 1 nonculture CSF infection test sent, 3 (2.6%; 95% CI, 0.5%-7.4%) of which had a positive result, a slightly higher proportion than the overall cohort (Table 3).

From a value perspective, if nonculture CSF infection testing on lumbar puncture specimens with normal cell counts was not performed in this cohort, we would have eliminated testing on 1270 lumbar puncture specimens, a total of 3021 of the 5054 total tests sent (59.7%, 95% CI, 58%-61%) of all nonculture CSF infection tests sent for this cohort. However, we would have missed the 19 positive test results in 18 children outlined in Table 3.

## Discussion

Lumbar puncture specimens with normal CSF cell counts were common in this cohort of immune-competent children but were rarely associated with nonculture CSF infection tests with a positive result and implications for clinical care. The proportion of these specimens sent for nonculture infection testing was lower compared with the proportion observed in those with elevated cell counts (38.1% vs 51.9%; *P* < .001), but this is only a 27% difference in magnitude, suggesting that cell counts alone are not a factor in the decision to send specimens for a nonculture CSF infection test in most children. Furthermore, once 1 nonculture infection test was sent for testing, both groups had a similar proportion of multiple tests sent (65.1% vs 67.1%), suggesting that once the decision is made to send for nonculture infection testing, CSF cell counts are not a factor in the number of tests ordered. Annual testing frequency has not substantially changed in the past 10 years at Children’s Hospital of Philadelphia. Seasonal testing frequency increases annually in July to September, which may be associated with seasonal enteroviral transmission (and subsequent increased testing practice) or with the relative inexperience of new house officers, as proposed in previous CSF PCR testing epidemiologic studies.^22^ Testing practices were similar in infants 6 to 12 months of age compared with testing in older children.

Eight of the 18 children with normal cell counts who had testing with a positive result (7 CSF EV PCR, 1 CSF HHV-6 PCR) in the cohort were discharged before the results were available. One child underwent a lumbar puncture as an outpatient, and the positive nonculture infection test result (CSF measles IgG) was believed to be associated with prior intravenous immunoglobulin and not clinically significant. One child had a positive CSF enterovirus PCR result that prompted an acyclovir sodium discontinuation. The 2 children with positive test results that required clinical intervention (1 CSF HSV PCR, and 1 CSF measles IgG) each had other clear clinical and neuroradiographic signs of infection. The remaining 6 children had positive results that did not affect their medical treatment.

Cell count results from CSF are typically available within hours, but other nonculture CSF infection tests can take days or weeks to complete. Implementing a testing strategy to delay the decision to send specimens for additional nonculture CSF infection testing until CSF cell counts were available could have reduced up to 60% of all nonculture CSF infection testing for this cohort. This type of change could reduce unnecessary diagnostic testing and medical costs, without substantially delaying the results in children with elevated cell counts or those with normal cell counts and a specific clinical concern based on other clinical data, thus improving value-based care.

Low-value care (or clinical care that provides little or no patient advantage, may cause harm, or may yield marginal positive outcomes at a disproportionately high cost) is believed to constitute up to one-third of health care costs in the United States.^23,24^ Identifying sources of low-value care and minimizing it are critical: small changes applied across populations can be associated with meaningful reductions in domestic health care expenditures.^23^

Several groups have previously examined various interventions to reduce unnecessary nonculture CSF infection testing in different populations. In a retrospective study of 732 adult CSF specimens submitted for testing at the Mayo Clinic, Tang et al^22^ demonstrated that restricting HHV PCR testing to specimens with CSF WBC count higher than 5 cells/μL and protein level greater than 45 mg/dL would have saved almost one-third of costs without reducing sensitivity. Hanson et al^25^ demonstrated that limiting HSV PCR testing to requests on specimens with CSF WBC count higher than 5 cells/μL or protein level greater than 50 mg/dL, specimens obtained from an immunocompromised individual (HIV positive or recipient of organ transplant) or from a child younger than 2 years, reduced testing by 22% (363 of 1659 specimens) and missed only 2 positive CSF HSV PCR results, which in retrospect did meet the criteria for testing. Hauser et al^26^ demonstrated that, among 6357 CSF HSV PCRs ordered on adults at a Veterans Affairs hospital over a 13-year period, no clinically significant positive results would have been missed by applying the Hanson et al^25^ criteria. In a split retrospective observational study with a screening and validation cohort, Wilen et al^27^ demonstrated that limiting the 8791 HSV, VZV, cytomegalovirus, and enterovirus PCRs ordered during the screening phase to immune-competent adults with CSF WBC count higher than 10 cells/μL would have reduced testing by about 55%, missing only 1 clinically significant result. In their validation cohort, 17 of 2126 tests were positive for HSV, VZV, cytomegalovirus, or enterovirus; applying the same criteria would not have missed any clinically significant test results.

This current study adds to the growing body of literature on CSF testing and low-value care. It provides pediatric data on diagnostic yield of nonculture CSF infection testing across a broad battery of common tests. Unlike the adult studies, protein levels were largely normal among the children in this cohort with positive nonculture CSF infection testing with the exception of 1 child (elevated to 49 mg/dL; Table 3).

Ultimately, the decision to send specimens for nonculture CSF infection testing requires a nuanced approach. It should incorporate community prevalence of infection, known pathogen risk factors, and patient-specific clinical judgment.

### Limitations

This study has several limitations. First, although we suspected that CSF cell counts were not available at the time the decision was made to perform nonculture CSF infection testing, we did not know the definite timing of clinical decision making for each lumbar puncture in this large cohort. For this reason, we excluded nonculture infection tests ordered on days after the lumbar puncture, to exclude tests that were ordered on the basis of preliminary CSF data. Second, given the large size of the cohort, we based the exclusion criteria on *International Classification of Diseases, Tenth Revision,* codes. During the manual EMR review for quality assurance, we discovered that some children with lumbar puncture procedures ordered for noninfectious concerns (eg, demyelinating lesions, cytologic testing, and hemorrhage) and for neurosurgical concerns (eg, lumbar drains, hydrocephalus status post shunt, and others) remained in the cohort, likely because the pertinent diagnosis had not yet been added to their EMR. These findings may imply that our conclusions are more generalizable to broader populations, but further focused study is warranted.

Third, the data we used were produced by EMR query, making it possible that some CSF tests ordered may have been missed. To minimize this risk, we manually reviewed all 3331 records of children with normal cell counts to ensure accurate documentation of nonculture CSF infection tests performed and their results. Fourth, we did not include infants younger than 6 months, limiting the study’s application to this population. This decision was a priori, given that the CSF WBC profiles of infants in the face of infection have substantial variability.^28,29^ Thus, the interpretation of CSF data in younger infants compared with older infants is a different and unique clinical challenge. Fifth, given the variation in CNS pathogen prevalence in different source populations, these data may not be completely generalizable to all tertiary care pediatric hospitals. However, the potential to reduce nonculture CSF infection testing in children with normal CSF cell counts should be applicable in any setting.

## Conclusions

This study’s findings suggest that lumbar puncture specimens with normal cell counts are common in immune-competent children older than 6 months but are rarely associated with nonculture CSF infection tests with positive results that are independently associated with clinical care. Implementing a hospitalwide intervention to delay the decision to order nonculture CSF infection testing, or to hold specimens in the laboratory until CSF cell counts are available, could dramatically reduce unnecessary testing and associated costs, improving value-based care. A nuanced approach is needed in the decision to send specimens for nonculture CSF infection testing. This approach should include community prevalence of infection, known pathogen risk factors, and patient-specific clinical judgment.

## Notes

Supplement. **eMethods.** Exclusion Criteria for *ICD-10* Codes
