# Supplementary material for: Assessment of Diagnostic Yield of Nonculture Infection Testing on Cerebrospinal Fluid in Immune-Competent Children
Source: JAMA Netw Open. 2019 Jul 19;2(7):e197307. doi: 10.1001/jamanetworkopen.2019.7307 (PMC6646983; doi:10.1001/jamanetworkopen.2019.7307)
Supplement: Supplement. — eMethods. Exclusion Criteria for ICD-10 Codes [file jamanetwopen-2-e197307-s001.pdf]

Supplementary Online Content

McGuire JL, Tuite NV, Swami SK, Avery RA. Assessment of diagnostic yield of nonculture infection testing on cerebrospinal fluid in immune-competent children. *JAMA Netw Open*. 2019;2(7):e197307. doi:10.1001/jamanetworkopen.2019.7307

eMethods. Exclusion Criteria for *ICD-10* Codes

This supplementary material has been provided by the authors to give readers additional information about their work.

©2019 McGuire JL et al. *JAMA Network Open.*

eMethods. Exclusion Criteria for *ICD-10* Codes

Indwelling CSF shunts/catheters were defined by ICD10 codes T85.739 and Z98.2. Oncologic conditions were defined by ICD10 codes C, D00, D01, D02, D03, D04, D05, D06, D07, D08, D09, D37, D38, D39, D40, D41, D42, D43, D44, D45, D46, D47, D48, D49, T86, Z08, Z85, Z94. Immunologic conditions were defined by ICD10 codes B20, D59, D60, D61, D69.41, D69.42, D70.0, D70.1, D70.2, D70.4, D70.8, D70.9, D71, D72.0, D75, D76, D80, D81, D82, D83, D84, D86, D89, I25.811, Z21. Rheumatologic conditions were defined by ICD10 codes K50, M04, M05, M06, M07, M08, M30, M31, M32, M33, M34, M35.0, M35.1, M35.2, M35.3, M35.4, M35.5, M35.6, M35.8, M35.9, M36, M45, M46. Exclusionary immune suppressing medications included monoclonal antibodies (adalimumab, alemtuzumab, certolizumab, crizotinib, daclizumab, gloimumab, infliximab, ixeekizumab, natalizumab, rituximab, tocilizumab, ustekinumab, vedolizumab) and other chemotherapeutic agents or immunomodulators (abatacept, anakinra, azathioprine, cyclophosphamide, cyclosporine, cytarabine, daunorubicin, doxorubicin, dimethyl fumarate, etanercept, etoposide, everolimus, filgrastim, fingolimod, fluorouracil, glatiramer acetate, interferon beta, leflunomide, leukovorin, mercaptopurine, methotrexate, mitoxantrone, mycophenolate, PEG-asparaginase, PEG-filgrastim, PEG-interferon, sirolimus, tacrolimus, teriflunomide, and vinblastine).

©2019 McGuire JL et al. *JAMA Network Open.*
